# The Interaction of Classical Complement Component C1 with Parasite and Host Calreticulin Mediates *Trypanosoma cruzi* Infection of Human Placenta

**DOI:** 10.1371/journal.pntd.0002376

**Published:** 2013-08-22

**Authors:** Christian Castillo, Galia Ramírez, Carolina Valck, Lorena Aguilar, Ismael Maldonado, Carlos Rosas, Norbel Galanti, Ulrike Kemmerling, Arturo Ferreira

**Affiliations:** 1 Institute of Biomedical Sciences, Faculty of Medicine, University of Chile, Santiago, Chile; 2 Department of Preventive Animal Medicine, Faculty of Veterinary Medicine, University of Chile, Santiago, Chile; Federal University of São Paulo, Brazil

## Abstract

**Background:**

9 million people are infected with *Trypanosoma cruzi* in Latin America, plus more than 300,000 in the United States, Canada, Europe, Australia, and Japan. Approximately 30% of infected individuals develop circulatory or digestive pathology. While in underdeveloped countries transmission is mainly through hematophagous arthropods, transplacental infection prevails in developed ones.

**Methodology/Principal Findings:**

During infection, *T. cruzi* calreticulin (TcCRT) translocates from the endoplasmic reticulum to the area of flagellum emergence. There, TcCRT acts as virulence factor since it binds maternal classical complement component C1q that recognizes human calreticulin (HuCRT) in placenta, with increased parasite infectivity. As measured *ex vivo* by quantitative PCR in human placenta chorionic *villi* explants (HPCVE) (the closest available correlate of human congenital *T. cruzi* infection), C1q mediated up to a 3–5-fold increase in parasite load. Because anti-TcCRT and anti-HuCRT F(ab′)_2_ antibody fragments are devoid of their Fc-dependent capacity to recruit C1q, they reverted the C1q-mediated increase in parasite load by respectively preventing its interaction with cell-bound CRTs from both parasite and HPCVE origins. The use of competing fluid-phase recombinant HuCRT and F(ab′)_2_ antibody fragments anti-TcCRT corroborated this. These results are consistent with a high expression of fetal CRT on placental free chorionic *villi*. Increased C1q-mediated infection is paralleled by placental tissue damage, as evidenced by histopathology, a damage that is ameliorated by anti-TcCRT F(ab′)_2_ antibody fragments or fluid-phase HuCRT.

**Conclusions/Significance:**

*T. cruzi* infection of HPCVE is importantly mediated by human and parasite CRTs and C1q. Most likely, C1q bridges CRT on the parasite surface with its receptor orthologue on human placental cells, thus facilitating the first encounter between the parasite and the fetal derived placental tissue. The results presented here have several potential translational medicine aspects, specifically related with the capacity of antibody fragments to inhibit the C1q/CRT interactions and thus *T. cruzi* infectivity.

## Author Summary

The *Trypanosoma cruzi* protozoan infects 9 million people in Latin America and increasing numbers in North America, Europe, Australia, and Japan. It is an important neglected parasitic disease in the Americas with no safe treatment available. One third of those infected develops incapacitating pathology. While in poor countries transmission of the parasite is mainly through blood feeding insects, transplacental infection is increasingly important in developed regions. Herein we show that *T. cruzi* calreticulin (TcCRT), a multifunctional protein, exteriorized by the parasite, mediates infection of human placenta, since it binds human complement component C1, a “danger signal” detector. (Complement is an innate immune defense system, with more than 40 plasma or membrane-bound proteins). However, in a parasite strategy, maternal C1 is utilized to infect placenta. Fetal calreticulin (HuCRT) is also easily detectable in placental tissues that are in direct contact with maternal blood. Thus, C1q by bridging parasite and HuCRT mediates high increases in cultured placental tissue infection with damaging consequences. Complete reversion of C1-mediated infection and a decreased placental damage, is observed in the presence of anti-TcCRT and anti-HuCRT antibody fragments, or fluid-phase competing HuCRT. It remains to be determined whether these mechanisms also operate in other intracellular protozoa.

## Introduction

*Trypanosoma cruzi* is the protozoan that causes Chagas' disease [1], an acute and chronic illness affecting 9 million people in Latin America [2] and causing 50,000 deaths per year [3]–[5]. Increasing numbers of infected people have been detected in North America, Europe, Australia, and Japan. Indeed, in the United States, more than 300,000 cases have been reported [4], [6]. It is one of the most important neglected parasitic diseases in the Americas and no safe treatment is available [6]. One third of those infected develops incapacitating circulatory or digestive pathology [4].

Pharmacological treatment of the infection, although effective in some cases, is complicated by the toxicity of the main drugs used (Nifurtimox and Benznidazole) [4], [7]. Therefore, identification and immune intervention on different molecular targets, such as those involved in *T. cruzi* infectivity and in the parasite capacity to inactivate the complement system, together with conventional pharmacological therapies, may result in synergic or even additive effects.

Several *T. cruzi* surface molecules promote infectivity. Among them gp82, gp30, gp35/50, trans-sialidase, gp85 and calcineurin B, are all metacyclic and tissue culture-derived trypomastigote surface molecules, with Ca^+2^ signal-inducing activities. They play important variable roles in the parasite attachment to host cells and invasion [8], [9].

*Trypanosoma cruz*i calreticulin (TcCRT), a 45 kDa protein [10], containing the KDEL-Endoplasmic Reticulum (ER) retention sequence [11], [12], translocates from the ER to the parasite exterior, and strongly inhibits the classical pathway of human complement activation [13]. It also inhibits angiogenesis [14] and tumor growth [15]. Most important, on the parasite surface TcCRT behaves as a potent virulence factor [16].

C1q plays an important role in the *in vitro T. cruzi* infection of macrophage and fibroblast cell lines, although the parasite and host cell receptors for the complement component were not identified [17]. We have shown that C1 interacts with CRT from parasite and human origins. Thus, TcCRT, differently from the other described parasite surface receptors involved in infectivity, interacts with complement component C1 and utilizes it as an adaptor molecule to recognize host cells [16], [18]. Thus, translocation of TcCRT from the ER to the membrane, not only inhibits the classical pathway of complement by interacting with C1 (q,r2,s2) [13], [19] but, in a parasite apoptotic mimicry effort, it also promotes infectivity, most likely by generating effective C1q-mediated “eat me” signals.

Attempts to interfere with the C1/TcCRT interactions with whole Igs or their F(ab′)_2_ fragments have opposite and predictable outcomes, both *in vitro* and *in vivo*. *In vitro*, in a cell-free system, TcCRT binds C1q, and whole IgG anti-TcCRT mediates Fc-dependent incorporation of additional C1q molecules onto the immune complexes, with likely consequent increased *in vivo* infectivity [16]. On the other hand, F(ab′)_2_ fragments from anti-TcCRT IgGs, devoid of their C1q-fixing Fc domains, revert the TcCRT/C1 interaction [16]. Thus, in mice, whole IgG anti-TcCRT and their F(ab′)_2_ fragments respectively stimulate and inhibit *T. cruzi* infectivity [14], [16].

Within the uterus, during mammalian gestation, a balance between tolerance to a hemiallogeneic fetus and protection against mother-borne pathogens must be operative. Subversion of this equilibrium by pathogens can complicate pregnancy or lead to vertical transmission of pathogens with fetal, perinatal or later morbidity or mortality [20]. The placenta is a chimeric organ made of maternal and fetal cells that nourishes and protects the fetus. Fetal derived invasive extravillous trophoblasts anchor the placenta in the uterine implantation site (decidua) and restructure maternal arteries to facilitate blood access to fetal derived syncytium villous trees [21].

The general consensus is that the syncytiotrophoblast (ST) is a formidable barrier to infection with microbes important during pregnancy. These properties of the ST derive from: i). Absence of intercellular junctions; ii). Absence of E-cadherin [22], [23]; iii). Presence of a network of profuse branched microvilli; iv). Presence of a dense cytoskeleton network [24]; v). A prevalence of an apical to basal directionality of nutrient transport [25] that may also preclude endocytic uptake of pathogens on the basal side; vi). Abundance of fused mitochondria [26] and, vii). ST production of reactive nitrogen species [27].

Despite the effectiveness of the placental barrier, mother-to-child transmission leading to congenital Chagas' disease and other adverse neonatal outcomes is increasingly recognized [28]–[31]. During pregnancy, the rate of vertical transmission of*T. cruzi* infection is approximately 5–10% (close to that reported for untreated HIV/AIDS [32]). There are over 14,000 cases of congenital Chagas' disease now reported in Latin America [33], with 2,000 newborns infected annually in North America alone, a situation also increasing in other developed countries [31].

We have previously shown that *T. cruzi* induces ST destruction and detachment in human placenta chorionic *villi* explants (HPCVE), together with selective disorganization of the basal lamina and of collagen I in the connective tissue of the villous stroma [34], as well as apoptosis [35], [36]. These effects may be mediated by cruzipain or metalloproteases that degrade the local extracellular matrix components such as collagen type I, IV and fibronectin [37]. Thus, the parasite overcomes the placental barrier and accesses the fetus [34], [37].

There are important differences between human and mouse placenta [38], [39] that limit the utility of these animals as experimental models for basic *in vivo* studies on congenital transmission of this infection; the main difficulties being the low yield of congenital transmission to the litter using different strains [40], [41] and the fact that murine placenta has a labyrinthine structure and the human placenta a villous one [42].

The use of HPCVE allowed us to propose that, because CRTs from parasite and fetal cell origins interact with maternal complement component C1, these three molecules strongly promote *T. cruzi* infection of human placenta. The results presented here agree with this proposal.

## Methods

### Bioethics statement

Written informed consent for the experimental use of the placenta was given by each patient. The protocols involving the use of human placenta were approved by the Ethics Committee for Research in Human Beings of the Faculty of Medicine, University of Chile (N° 041-2011) and by the Committee for Bioethics from The National Council of Scientific and Technologic Research (CONICYT-Chile).

The use of rabbits for the generation of antibodies has been described previously [43] and followed the Guide for the Care and Use of Laboratory Animals of the National Institutes of Health, U.S.A. The protocols were approved by the Bioethics Committee, Faculty of Medicine, and University of Chile and by The National Council of Scientific and Technologic Research (CONICYT-Chile). Animals were maintained in our Central Experimental Animal Facility and were cared for by trained personnel and veterinarians.

### Human placenta and chorionic *villi* explants

Human term placentas were obtained from uncomplicated pregnancies from vaginal or caesarean delivery from the Maternity Section, Hospital San José, Santiago, Chile. Exclusion criteria for patients were any maternal, fetal or placental pathology. Placentas were collected in cold PBS and processed no more than 30 min after delivery. Their maternal and fetal surfaces were discarded and villous tissue was obtained from the central part of the cotyledons. HPCVE were washed with PBS in order to remove blood, cut in approximately 0.5 cm^3^ pieces and co-cultured with infective Y strain trypomastigotes (2×10^4^/ml) for 2 h in 1 ml of RPMI. In order to determine the roles of TcCRT, fetal HuCRT and maternal C1q (the complement moiety deprived of serine proteases C1r and C1s activities), in *T. cruzi* infectivity in HPCVE, the explants were co-cultured with combinations of the following reagents: i). C1q (Complement Technologies, Taylor, Texas, USA), ii). IgG polyclonal antibodies anti-recombinant TcCRT [43] or anti-recombinant HuCRT [44], both antisera generated in rabbits in our laboratories, by conventional methodology. Each antisera was tested in western blottings against the homologous and orthologous recombinant antigens, and also against a wild type *E. coli* extract. As usually occurs with polyclonal antisera, only at high concentrations, both of them marginally recognized the orthologous antigen. No antigen recognition was evident in *E. coli* extracts [43], iii). F(ab′)_2_ IgG fragments anti-recombinant TcCRT or anti-recombinant HuCRT, generated by pepsin digestion [16], [43] and, iv). Fluid phase recombinant HuCRT. (DNA coding for HuCRT was kindly donated by Prof. Wilhelm Schwaeble, Leicester University, UK, and was expressed and purified in our laboratory). HPCVEs were incubated with trypomastigotes in RPMI supplemented with HIFBS (control) and C1q, in the presence of HuCRT, in solution. All these reagents were tested in several concentrations, in the different experimental groups, as indicated in the Results section. Basal infectivity was obtained when HPCVE were incubated with trypomastigotes supplemented with heat-inactivated fetal bovine serum (HIFBS). The amount of parasite DNA in HPCVE was determined by qPCR. Experiments shown are representative of those performed in three different placentas.

### *T. cruzi* DNA amplification by real time PCR (qPCR)

Genomic DNA was extracted from the placental tissue with the Wizard Genomic DNA Purification Kit (Promega, USA), according manufacturer's instructions and quantified by μDrop Plate DNA quantification system in a Varioskan Flash Multimode Reader (Thermo Scientific, USA). For amplification of human and parasite DNA, two specific primer pairs were used. A 100 bp human GAPDH sequence was amplified using the primers hGDH-F (50-TGATGCGTGTACAAGCGTTTT-30) and hGDH-R (50-ACATGGTATTCACCACCCCACTAT-30), designed using the Primer Express software (version 3.0; Applied Biosystems). For *T. cruzi* DNA detection a 182 bp sequence of satellite DNA was amplified by using TCZ-F 50-GCTCTTGCCCACAMGGGTGC-30 and TCZ-R 50-CAAGCAGCGGATAGTTCAGG-30 primers [45]. Relative quantification analysis of the results was expressed as RQ value by the comparative Control (ΔΔCt) method [46].

### Histological and immunohistochemical techniques

The placental *villi* were fixed in 10% formaldehyde in 0.1 M phosphate buffer (pH 7.3) for 24 h, then dehydrated in alcohol, clarified in xylene, embedded in paraffin, and sectioned at 5 µm (Microtome Leitz 1512). Paraffin histological sections were stained with hematoxylin-eosin for routine histological analysis. In order to detect fetal and maternal CRT in human placenta tissues, standard immunohistochemical techniques were used. Briefly, histological sections were treated with 3% hydrogen peroxide in methanol for 10 minutes and incubated for 30 minutes in Dako Target Retrieval (Dako, Carpinteria, CA, USA) on a steamer. The tissue was probed with rabbit anti-human CRT IgG [43], followed by goat anti-rabbit IgG conjugated to peroxidase and then a commercial substrate (Histomouse MAX-AEC Broad SpectrumTM Kit (Invitrogen, Camarillo, CA, USA) employed, for staining of CRT.

### Statistics

Results are expressed as mean and SDs. The significance of differences was evaluated using ANOVA followed by Dunnett's post-test. The control group corresponds to HPCVE incubated with culture media supplemented with heat-inactivated FBS and infected with the parasite in the absence of any of the molecules tested.

## Results

The roles of TcCRT, fetal HuCRT and maternal C1q, in *T. cruzi* infectivity in HPCVE were investigated. Combinations of C1q, rabbit anti-TcCRT or HuCRT IgG or their F(ab′)_2_ fragments, or fluid phase recombinant HuCRT were tested in the different experimental groups. Basal infectivity was obtained when HPCVE were incubated with trypomastigotes supplemented with HIFBS. The amount of parasite DNA in HPCVE was determined by qPCR.

### C1q mediates *T. cruzi* infectivity in HPCVE

**Figure 1:**
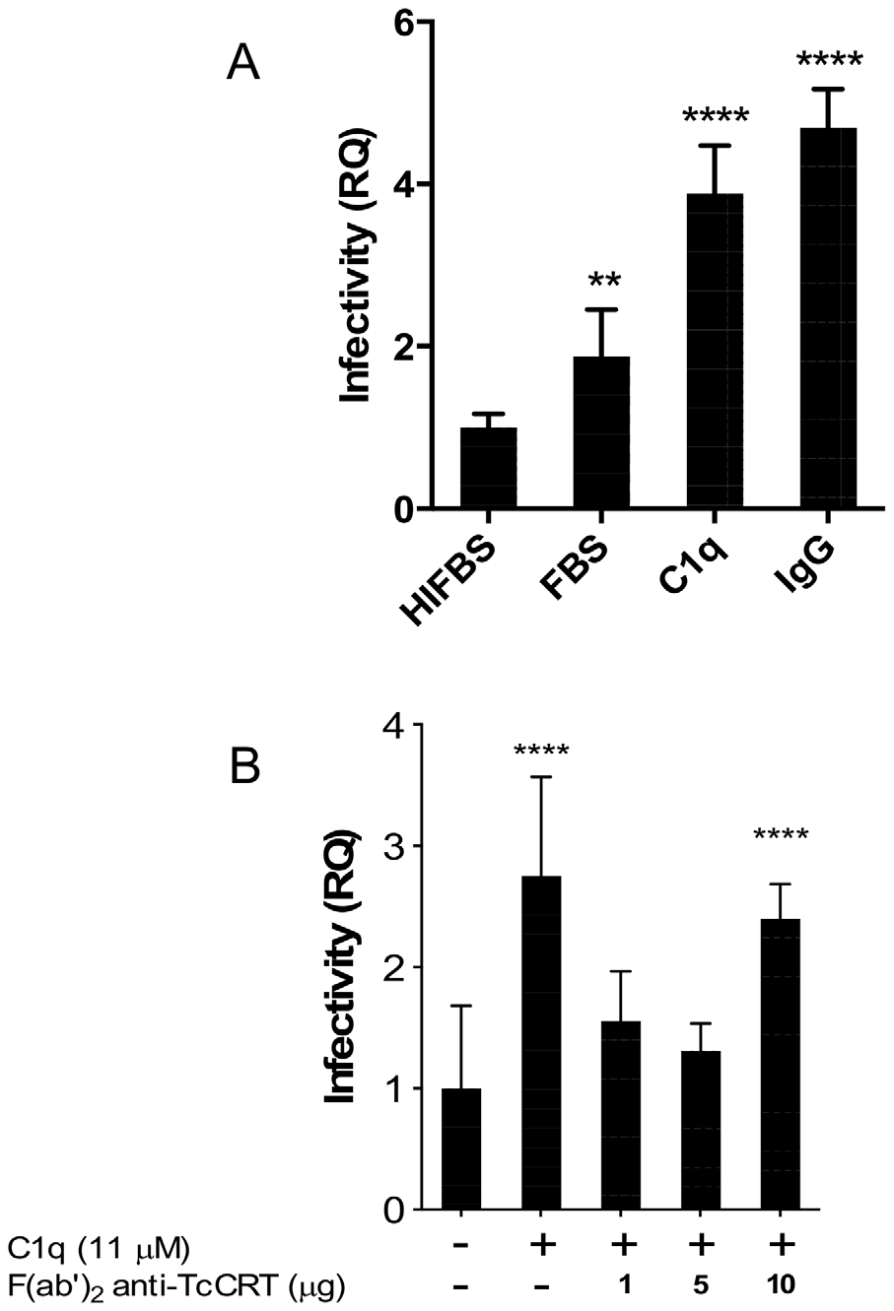
C1q increases *T. cruzi* infectivity in HPCVE, which is prevented by F(ab′)_2_ bivalent anti-TcCRT IgG fragments. The explants were incubated with trypomastigotes, in RPMI, alternatively supplemented with FBS, HIFBS, C1q (11 µM) or C1q plus IgG (50 µg/ml) anti-TcCRT (A). In (B), the explants were incubated with trypomastigotes, in RPMI supplemented with HIFBS and C1q, in the presence of F(ab′)_2_ antibody fragments anti-TcCRT. The amount of parasite DNA in HPCVE was determined by qPCR. Data represent the mean of 5 observations and their standard deviations. The significance of differences was evaluated using ANOVA followed by Dunnett's post-test. **p≤0.01, ****p≤0.0001.

To address whether C1q (the complement moiety deprived of serine proteases C1r and C1s activities) is involved in the infection of HPCVE, explants were incubated with trypomastigotes, in the alternative presence of C1q or C1q plus whole IgG anti-TcCRT. Increases in parasite DNA of 4.5, and 6.0 fold, were respectively observed, as compared to the basal infection in the presence of HIFBS (Figure 1A). Fc-dependent binding of additional C1q molecules could explain why the presence of polyclonal whole IgGs anti-TcCRT increased parasite infectivity [16]. When FBS was used, infectivity was moderately increased most likely because of the presence of residual active C1q, or other non-identified factor(s). For comparison purposes infectivity in the presence of HIFBS was considered as basal and used as control in all subsequent experiments.

### C1q mediates *T. cruzi* infectivity in HPCVE, by virtue of its capacity to interact with TcCRT

Since, in order to promote infectivity, C1q binds to trypomastigote translocated CRT, this interaction should be prevented by bivalent TcCRT-binding antibody fragments deprived of their Fc domains and hence unable to bind C1q. Thus, in HPCVE incubated with trypomastigotes, in the presence of C1q and F(ab′)_2_ polyclonal antibody fragments anti-TcCRT, the C1q-dependent parasite ability to infect placental cells was reverted to basal levels (Figure 1B).

### Human placenta expresses high levels of HuCRT at the ST level

**Figure 2:**
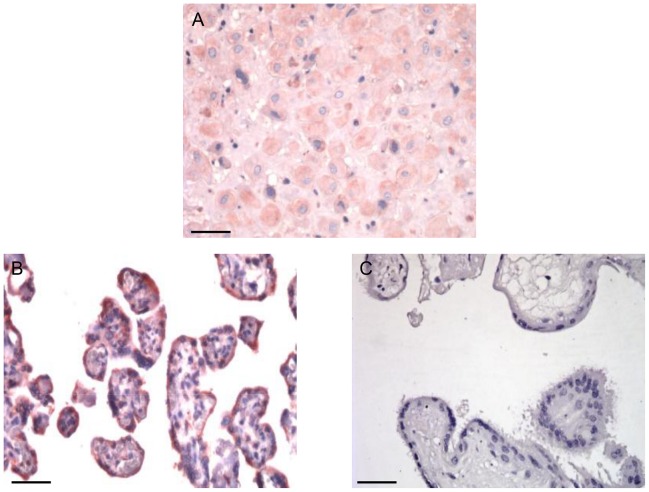
Human placenta expresses HuCRT, especially at the ST level. By immunohistochemistry the HuCRT is evident in human placenta as detected in a reddish tonality, by a polyclonal antiserum, at the basal maternal decidua (A) and free fetal *villi* (B). (C) Preimmune serum. Scale bars: 10 µm (A), 25 µm (B, C).

Human placental tissues are known to express high levels of HuCRT [47]. In order to define if ST expresses this molecule, as a possible receptor for TcCRT-bound C1q on the parasite surface, we compared HuCRT expression at the basal maternal decidua and free fetal *villi*. Polyclonal antibodies against HuCRT readily detected the human chaperone molecule mainly on fetal placenta *villi* ST, with a distribution consistent with its exposure on the ST surface (Figure 2).

### C1q mediates *T. cruzi* infectivity in HPCVE, because it interacts with fetal CRT on placental tissue

**Figure 3:**
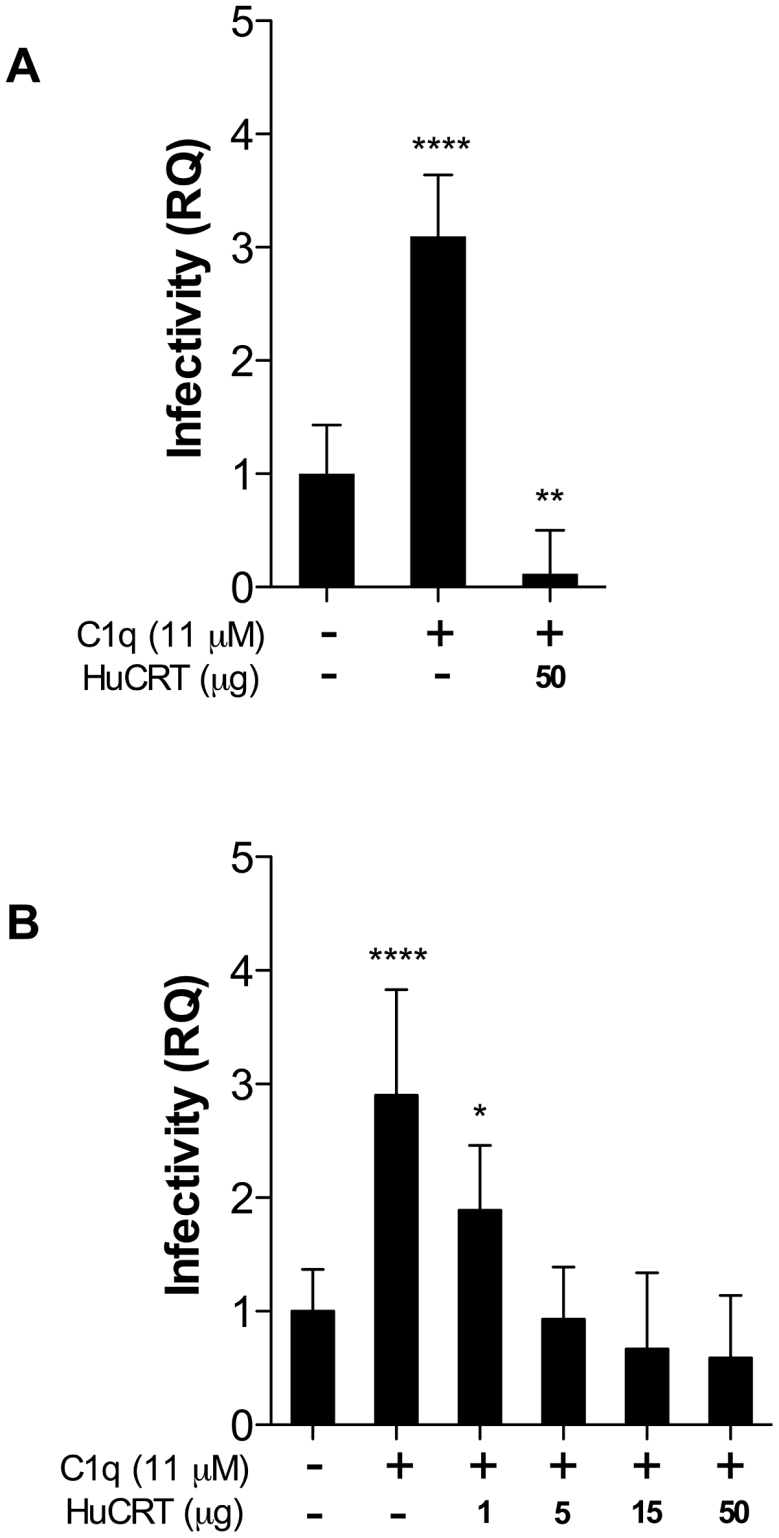
Fluid-phase HuCRT competes with parasite-bound C1q for binding to HPCVE. Explants were incubated with trypomastigotes in RPMI supplemented with HIFBS (control) and C1q in the presence of HuCRT (A). A fluid-phase HuCRT dose-dependent capacity to compete with *T. cruzi* infection of HPCVE is shown in (B). Data represent the mean of 5 observations and their standard deviations. The significance of differences was evaluated using ANOVA followed by Dunnett's post-test. *p≤0.05, **p≤0.01, ****p≤0.0001.

**Figure 4:**
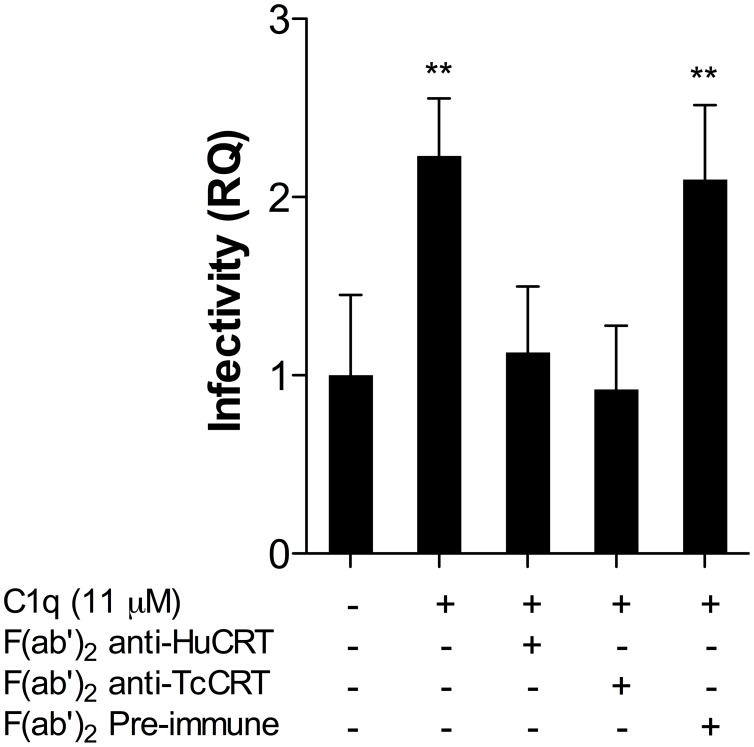
Both F(ab′)_2_ anti-HuCRT and anti-TcCRT bivalent antibody fragments revert C1q-mediated infectivity in HPCVE. Explants were incubated with trypomastigotes, in RPMI supplemented with HIFBS and C1q, in the presence of F(ab′)_2_ anti-HuCRT or anti-TcCRT IgG fragments. As negative controls, F(ab′)_2_ IgG fragments, obtained from preimmune sera, were used. All F(ab′)_2_ fragments were used at 50 µg/ml. The amount of parasite DNA in HPCVE was determined by qPCR. Data represent the mean of 5 observations and their standard deviations. The significance of differences was evaluated using ANOVA followed by Dunnett's post-test. **p≤0.01.

We then aimed at defining whether HuCRT participates as a possible receptor for the C1q/TcCRT complex present on the parasite surface. HPCVE were incubated with trypomastigotes, in the presence of C1q. In Figure 3A fluid-phase HuCRT inhibits even the basal infection (down to 12% of the control), most likely by competing with residual bovine C1q present in the HIFBS. In this experiment the C1q-mediated infectivity again reaches 4 times over the control (Figure 1A). Figure 4B summarizes the results of a fluid-phase HuCRT dose-response capacity to inhibit C1q-mediated HPCVE *T. cruzi* infection. Complete dose-dependent blocking of the C1q-mediated parasite ability to infect placental cells, is observed. In other words, in the absence of fluid-phase HuCRT, C1q mediates 6–30-fold increase in the *T. cruzi* capacity to infect HPCVE (Figure 3 A–B).

### C1q-mediates infectivity in HPCVE because of its capacity to interact with both, human and *T. cruzi* CRT

As an infectivity promoter, C1q should simultaneously bind to both HuCRT, on placental ST, and TcCRT, on the parasite. Concurringly, F(ab′)_2_ antibody fragments, derived from polyclonal IgG generated against recombinant CRTs from both human and parasite origins, completely reverted the C1q-mediated *T. cruzi* infectivity in HPCVE. The presence of F(ab′)_2_ IgG fragments, obtained from preimmune sera did not alter the infectivity mediated by the complement component (Figure 4).

### Inhibition of C1q- and HuCRT- mediated *T. cruzi* infectivity partially prevents histopathological alterations in HPCVE

**Figure 5:**
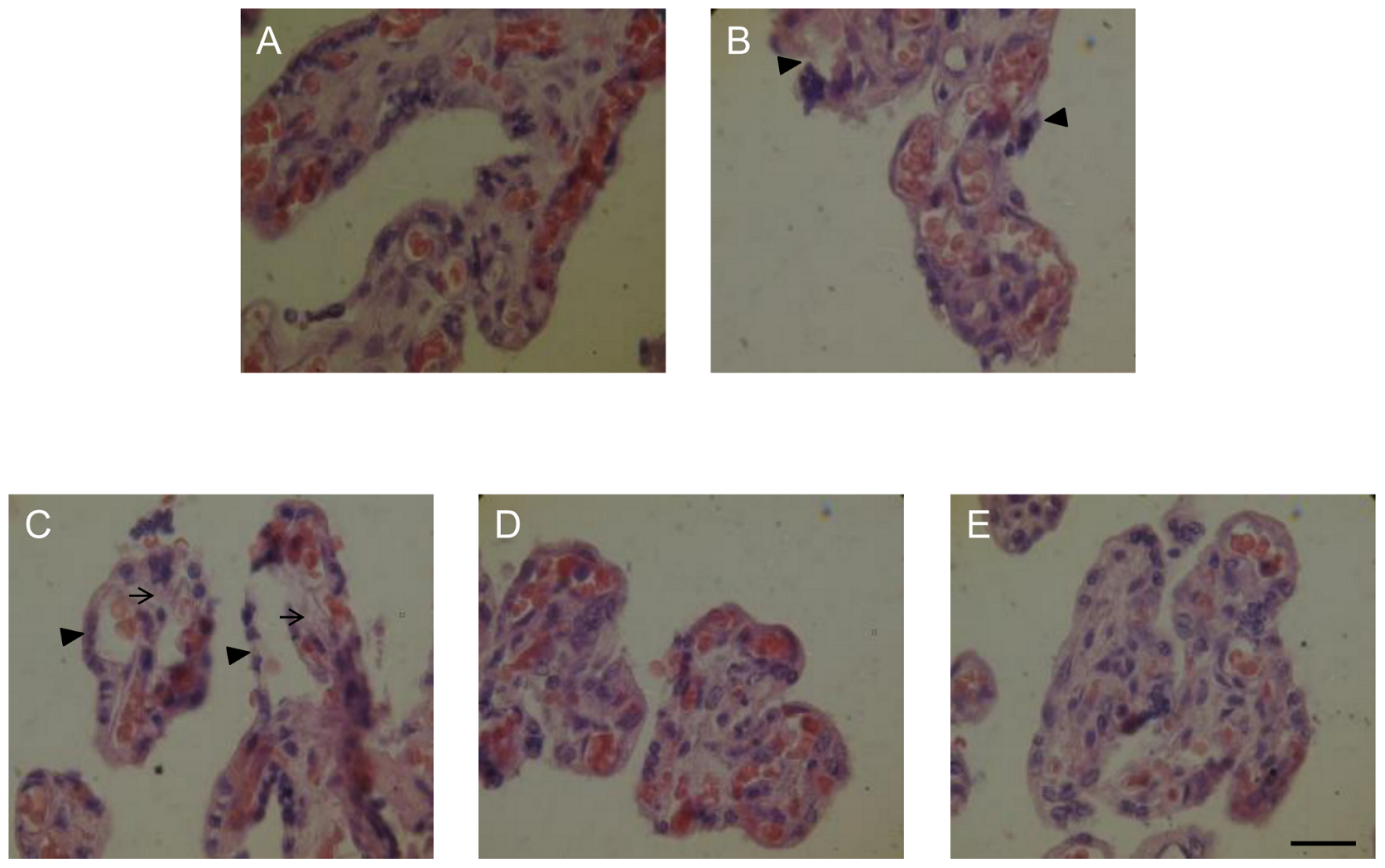
F(ab′)_2_ IgG fragments anti-HuCRT and fluid-phase HuCRT partially prevent C1q-mediated HPCVE histopathological alterations. In explants, incubated with trypomastigotes, in RPMI supplemented with HIFBS (B), a slight detachment of trophoblast is observed (B arrowhead), as compared to non-infected control (A). C1q (11 µM) mediated a more severe detachment of the trophoblast and destruction of the fetal connective tissue (C, arrowheads and arrows, respectively), that is partially prevented by F(ab′)_2_ IgG fragments anti-HuCRT (50 µg/ml) (D), or by fluid-phase HuCRT (5 µg/ml) (E). Tissues were processed by conventional histological procedures and stained with hematoxilin-eosin. Bar: Scale bar: 25 µm.

*T. cruzi* infection induces ST detachment in chorionic *villi*, together with disorganization of the basal lamina and of collagen I in the connective tissue [34]. Since the TcCRT/C1q/HuCRT interaction is involved in infectivity, we asked whether these interactions could be intervened, with consequent improvement of the histopathological alterations. Figure 5 summarizes the results obtained when HPCVE were incubated with trypomastigotes in RPMI, supplemented with HIFBS, where a slight detachment of trophoblast from the basal lamina is observed (Figure 5B, arrowheads), as compared with the non-infected control (Figure 5A). In the presence of exogenous human C1q, a strong trophoblast detachment (arrowheads) and destruction of fetal connective tissue can be observed (Figure 5C, arrows). Blocking of HuCRT by F(ab′)_2_ IgG fragments anti-HuCRT (Figure 5D), or blocking parasite-attached C1q with soluble HuCRT, results in partial prevention of trophoblast detachment (Figure 5E).

## Discussion

The placental barrier is effective to protect the fetus from mother borne microorganisms [48], but *T. cruzi*, like several other pathogens, has developed means to trespass it [49]–[57] and the relative relevance of congenital transmission of this infection is increasing [58], [59]. Several *T. cruzi* surface molecules promote infectivity [8], [9], however their association with *T. cruzi* placental infection has not been established. Efforts to define molecular mechanisms explaining how *T. cruzi* trespass the placental barrier are thus important.

We have proposed that among the many functions that parasite exteriorized TcCRT displays in the host/parasite interplay [18], of central relevance is its capacity to interact with classical complement component C1q. Based solely on this property, TcCRT behaves as a main virulence factor [16]. On the host cell side, fetal cC1qR (membrane-bound CRT acting as a receptor for the collagenous C1q tails) [60] interacts with TcCRT-bound maternal C1q and thus with the parasite.

Based on the known capacity of C1q to interact with Fc domains of IgG [16], attempts to interfere with the C1/TcCRT interactions with whole Igs or their Fc-deprived F(ab′)_2_ fragments have drastically different predictable outcomes, both *in vitro* and *in vivo*. *In vitro*, in a cell-free system, TcCRT binds C1q and F(ab′)_2_ fragments from anti-TcCRT IgGs, devoid of their C1q-fixing Fc domains, revert this interaction [43]. Most important, although pretreatment of trypomastigotes with C1q increased infectivity in the RAW murine cell line, as well as mice mortality and parasitemia, the F(ab′)_2_ fragments anti-TcCRT significantly interfered with the C1q-dependent infectivity [16].

As a consequence of infection, several phenomena that may facilitate, at least partly, the parasite progress towards fetal tissues have been described: i). Disassembly of cortical actin cytoskeleton [59], [61], ii). Presence of parasite DNA [62] and antigens [34] on the fetal side of placenta, iii). ST destruction and detachment in the chorionic *villi*, disorganization of the basal lamina and collagen I in the connective tissue of the villous stroma (VS) [35], [36], iv). Apoptosis in chorionic *villi*, especially at the ST and cytotrophoblast [35], v). Parasite cruzipain degradation of extracellular matrix (ECM) collagen type I, IV and fibronectin [63] and, vi). Endogenous ECM metalloproteases - mediated tissue damage [36], [64], [65]. These alterations occur after parasites contact with the HPCVE. Most likely, on the parasite side, translocated TcCRT and, on the maternal side, C1q and fetal HuCRT (cC1qR), all play important roles in the early first contact between trypomastigotes and placental tissue. This will be followed by an elaborate infective process, as described above.

There are precedents of *in vitro* or *ex vivo* systems to study *T. cruzi* infectivity of placental tissues [34]–[36], [62], [66]. Two constraints to these models have been posed in the literature [67]: i). The parasite DNA on the fetal side of placenta may correspond to debris transferred from the maternal side and, ii). The frequent use of large numbers of parasites (*i.e*. 10^5^–10^7^ for a few mg of chorionic *villi*), is highly superior to what can be expected in chronically infected women with parasitemias below 15 pg/ml.

HPCVE assays have been recognized as valid correlates not only to study the tissue damage caused by *T. cruzi* during placenta infection, but also to explain the earlier stages of vertical transmission [62], [68], [69]. First, damage to the tissue was evident when 2×10^4^ parasites were used per each HPCVE assay (Figure 5) and the parasite presence in chorionic *villi* is also microscopically clear in a similar experimental set up [34]. (This type of damage has also been reported for cytomegalovirus [70], *Plasmodium falciparum* [71] and *Toxoplasma gondii* [72]. Second, with regard to the large number of parasites used in other reported assays, we calibrated conditions for HPCVE infection down to 2×10^4^ trypomastigotes per each explant with adequate signals for parasite DNA detection in qPCR. This parasite concentration is within the range of expected parasite numbers reaching the placenta of infected women, in a 24 hour period [34].

Although it could be proposed that detachment and destruction of the infected tissue that interacts with the maternal blood, is a mechanism to avoid congenital infection [21], this mechanism is not efficient enough to mediate sterile fetal protection.

In this report we aimed at defining whether the above *T. cruzi* - infection promoting mechanisms are operative at the placental level. In HPCVE *ex vivo* assays, at least a 3–4 fold increase in parasite infectivity (average of the five repetitions shown in Figure 1–3) was mediated when both TcCRT and C1q were present. The infection-promoting capacity of FBS and, to a lesser extent, of HIFBS, could be respectively explained by the presence of putative bovine C1q, its active remnants, or other non-identified factor(s). For these reasons, and for comparison purposes, infectivity in the presence of HIFBS was considered as basal and used as control in all subsequent experiments. In placenta, Fc-dependent binding of additional exogenous C1q molecules could explain why the presence of polyclonal IgGs anti-TcCRT also mediated increased parasite infectivity (Figure 1A) [16].

We then reasoned that since, in order to promote infectivity, C1q must bind to parasite translocated TcCRT, this interaction should be interfered by F(ab′)_2_ polyclonal antibody fragments anti-TcCRT. Accordingly, complete reversion of C1q-dependent infectivity to basal levels was observed (Figure 1B).

Teleologically, it could be proposed that, during the co-evolution of the host/parasite interplay, the parasite did develop ways to subvert the humoral immune response against TcCRT. Indeed, if not all, most of humans infected with *T. cruzi* have IgGs anti-TcCRT in their plasma [73] and, in an apparent paradox, immunization of mice with TcCRT increases parasite infectivity [16]. Moreover, treatment of parasites with whole IgGs anti-TcCRT increases their capacity to infect murine macrophages *in vitro*. Opposite results are obtained *in vivo* when the corresponding F(ab′)_2_ IgG fragments were passively administered to infected animals, or *in vitro* when the parasites were treated with these modified antibodies [16].

High levels of HuCRT are known to be expressed in human placental tissues [47]. In order to define if this molecule is expressed at the ST, as a possible receptor for C1q bound to TcCRT on the parasite surface, we compared CRT expression at the maternal basal decidua and in fetal free *villi* (Figure 2B). HuCRT from maternal and fetal origins were respectively detected at both the decidua and *villi*. In *villi*, intense reactivity was detected at the ST, with a distribution consistent with a possible exposure on the ST surface. At the *villi* stroma the reactivity was weak (Figure 2B). It is thus likely that this fetal CRT may serve as a receptor for C1q already bound to the parasite by means of TcCRT. In other words, we propose that TcCRT-C1q complexes remain on the parasite surface and that C1q bridges the parasite with the ST, as a preamble to infection. This possibility was tested by incubating HPCVE with trypomastigotes, in the simultaneous presence of C1q and increasing concentrations of fluid-phase HuCRT. Complete dose-dependent blocking of the C1q-mediated parasite ability to infect placental cells, is observed. In other words, it is highly likely that fetal HuCRT, at the ST level, binds maternal C1q already bound to TcCRT on the parasite surface (Figure 3).

Moreover, the previous findings were further corroborated by showing that F(ab′)_2_ antibody fragments, derived from polyclonal IgG generated against recombinant CRTs from both human and parasite origins, completely and specifically reverted the C1q-mediated *T. cruzi* infectivity in HPCVE (Figure 4). Therefore, these antibody fragments effectively blocked the capacities of both ST fetal and parasite CRTs from binding C1q. Thus the infectivity mediated by this complement component was neutralized by these modified antibodies.

About 50% overall homology exists between TcCRT and HuCRT (up to 70% in some domains) [12]. However, at high concentrations, marginal cross reactivity of the anti- TcCRT antibodies with HuCRT and *vice versa* is observed in ELISA and IWB assays (not shown). However, this does not affect our conclusion with regard to CRT (from host and parasite origins) involvement in infectivity in HPCVE. The use of fluid phase HuCRT to inhibit infectivity, in a dose-dependent manner (Figure 3B), additionally supports this proposal.

Consistent with the notion that the parasite CRT/maternal C1q/fetal CRT interactions are involved in infectivity, when HPCVE were incubated with trypomastigotes in RPMI, supplemented with HIFBS, slight detachments of trophoblast from the basal lamina are observed, as compared with the non-infected control. When exogenous human C1q was present, trophoblast detachment was more evident. Blocking fetal CRT by F(ab′)_2_ IgG fragments anti-HuCRT, or blocking parasite-attached C1q with soluble HuCRT, resulted in partial prevention of trophoblast detachment (Figure 5). This is most likely due to decreased parasite penetration into the *villi* tissue and associated reported damages [34].

Since both Ficolins and MBL also interact with CRT, from human and parasite origins [13], [74], they will also probably facilitate parasite infectivity in this experimental set up. Since the “danger” signals detected by these components are different from IgGs, both whole IgGs anti-TcCRT as well as their F(ab′)_2_ fragments should inhibit infectivity mediated by these lectin pathway complement components.

*In vivo* models to validate our *ex vivo* results are complex to implement. Although the murine model, has several advantages to study congenital diseases (short pregnancy, large litters, short weaning time), the rate of *T. cruzi* vertical transmission to the fetuses is extremely low [75]–[77] and the structure of murine placenta is very different [38], [39].

TcCRT is a virulence factor as originally proposed by us [16], [18]. Recently, TcCRT has also been involved in the binding of thrombospondin-1 (TSP-1), with enhanced infectivity of mouse fibroblasts [78]. Differently from C1q (and possibly from Ficolins and MBL), TSP-1 is an ubiquitously located molecule, capable of interacting with a wide array of cellular proteins [79]. TSP-1 is expressed in fetal villous tissue [80] and could explain, at least partly, the basal *T. cruzi* infectivity obtained when HPCVE were incubated with trypomastigotes, in the absence of exogenous C1q (Figures 1; 3–4).

**Figure 6:**
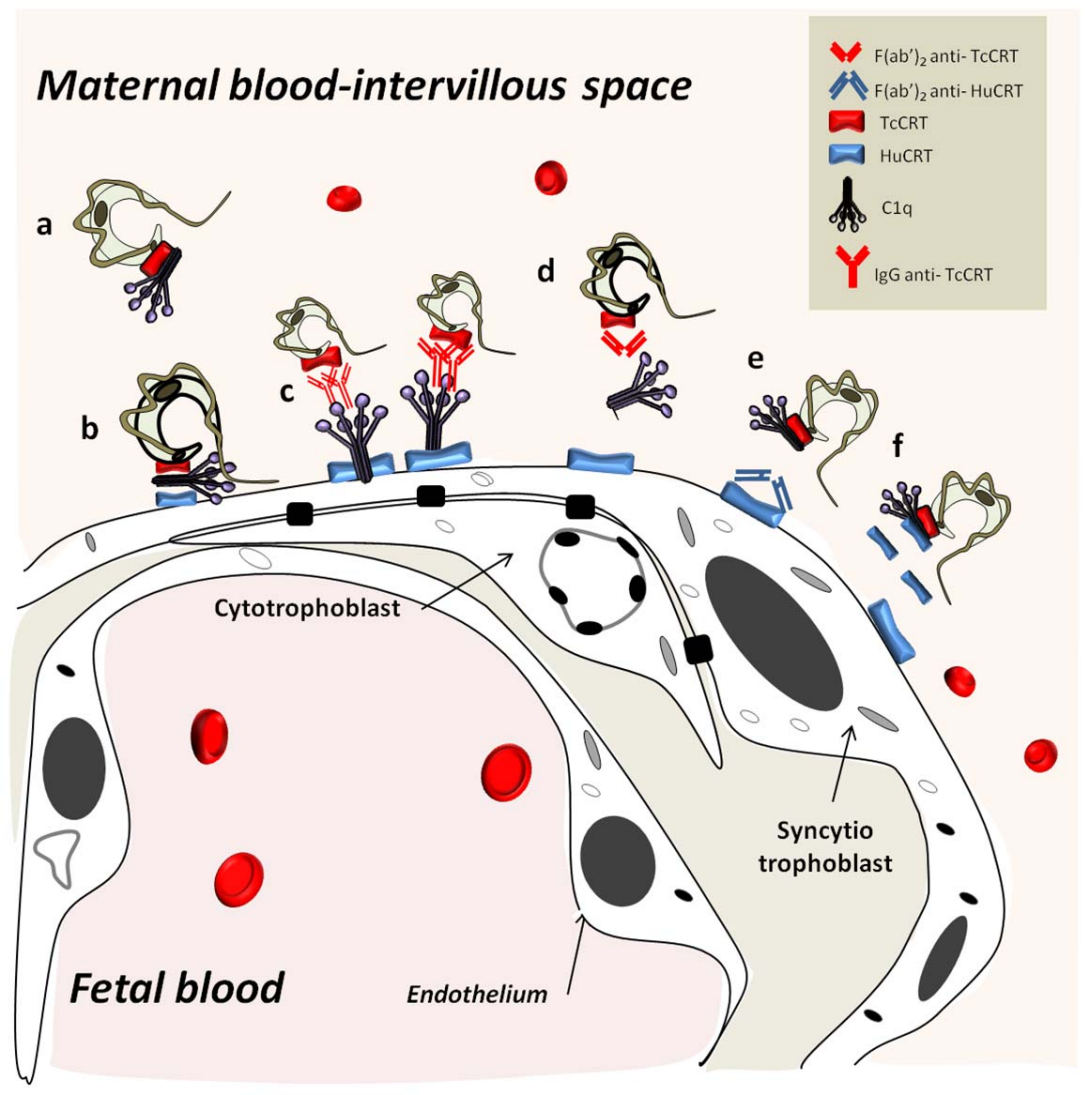
TcCRT, HuCRT and C1q participate in parasite infectivity in human placenta *in vivo*. C1q bound to translocated TcCRT in circulating infective trypomastigotes (a) bridges the parasite with a ST cell through HuCRT (b). Whole IgG anti-TcCRT promotes additional C1q binding and thus increased infectivity (c). The parasite interaction with ST is inhibited by F(ab′)_2_ IgG fragments anti-TcCRT (d) or anti-HuCRT (e). Fluid-phase HuCRT should compete with HuCRT on the ST thus preventing the parasite binding to ST (f).

Considering the previous results altogether, in Figure 6 we propose a simplified integrated model on the participation of maternal C1q and fetal CRT, on the one side, and parasite CRTs, on the other, in *T. cruzi* infection of human placenta. Most likely, *in vivo*, infective *T. cruzi* trypomastigotes circulate with maternal C1q already bound to translocated TcCRT. The results presented here have several potential translational medicine aspects, specifically related with the capacity of antibody fragments to inhibit the C1q/CRT interactions and thus *T. cruzi* infectivity.

Finally, based on these observations, it could be proposed that in pre immunized mothers, carrying whole anti-TcCRT antibodies, the parasite would be in a better position to infect the fetus. This could be a frequent event, given the high prevalence of anti-TcCRT antibodies in infected humans [73].
